# The impact of street greenery on active travel: a narrative systematic review

**DOI:** 10.3389/fpubh.2024.1337804

**Published:** 2024-02-28

**Authors:** Jiahua Yu, Hao Zhang, Xinyang Dong, Jing Shen

**Affiliations:** Department of Physical Education, China University of Geosciences (Beijing), Beijing, China

**Keywords:** street greenery, green space, walking, bicycling, active travel, review

## Abstract

**Background:**

Street greenery may have a profound effect on residents’ active travel (AT), a mode of transportation involving walking and cycling. This study systematically reviewed the scientific evidence on the effects of street greenery on active travel.

**Methods:**

A comprehensive search was performed using keywords and references in PubMed, Web of Science, Scopus, and Cochrane Library. The review included studies that met the following criteria: (1) Study design: experimental studies, cross sectional studies, (2) Participants: individuals of all ages, (3) Exposure variables: street greenery, including street vegetation (e.g., trees, shrubs, and lawns), (4) Outcomes: active travel behaviors (walking, cycling), (5) Article type: peer-reviewed articles, (6) Search time window: from the inception of relevant electronic literature database until 21 June 2023, (7) Geographic scope: worldwide; (8) Language: articles in English.

**Results:**

Twenty-six cross-sectional studies met the inclusion criteria and were analyzed. These studies employed objective metrics for assessing street greenery and varied methodologies to measure AT, including 14 using subjective measurements (like self-reported surveys), 10 using objective data (such as mobile app analytics), and two studies combined both approaches. This review identifies a generally positive impact of street greenery on active travel in various aspects. However, the extent of this influence varies with factors such as temporal factors (weekdays vs. weekends), demographic segments (age and gender), proximity parameters (buffer distances), and green space quantification techniques. Street greenness promotes active travel by enhancing environmental esthetics, safety, and comfort, while also improving air quality, reducing noise, and fostering social interactions. In addition, the study suggests that variables like weather, seasonality, and cultural context may also correlate with the effectiveness of street greenery in encouraging active travel.

**Conclusion:**

Street greenery positively influences active travel, contributing to public health and environmental sustainability. However, the findings also indicate the need for more granular, experimental, and longitudinal studies to better understand this relationship and the underlying mechanisms. These insights are pivotal for urban planners and policymakers in optimizing green infrastructure to promote active transportation, taking into account local demographics, socio-economic factors, and urban design.

## 1 Introduction

Active travel refers to a mode of transportation that primarily involves physical activities such as walking and cycling during leisure activities or commuting. This modality offers multifaceted health benefits, including the mitigation of chronic disease prevalence, reduction in premature mortality, and alleviation of depression risk (1, 2). Beyond personal health, active travel yields substantial environmental advantages by diminishing air pollution and easing traffic congestion, thereby contributing significantly to environmental preservation (3–6). Furthermore, it enhances social cohesion by fostering community interactions, accruing extensive societal advantages (7, 8).

Although there exists a wide consensus on the benefits of active commuting, the prevalence of bicycle usage in developing countries like China has rapidly declined over recent decades. This decline is linked to a constellation of factors, including rapid urbanization, technological progress, and lifestyle shifts (9). The configuration of the urban environment exert a profound influence on patterns of active travel (10). Urban design elements, including the configuration of streets, the presence of sidewalks, and the availability of safe and comfortable pathways, are pivotal in shaping individuals’ decisions to engage in walking or cycling (11). In this vein, street greenery emerges as an integral element of urban green infrastructure, significantly contributing to the visual appeal of urban landscapes (12). Its role in encouraging active travel has gained recognition, drawing considerable scholarly interest (13). Consequently, many cities in various countries have been channeling investments into the enhancement and upkeep of green spaces, aiming to elevate residents’ quality of life (14).

Street greenery, which includes the integration of vegetation such as trees, shrubs, lawns, and green walls into the streetscape, enhance the esthetic and functional appeal of urban thoroughfares (15). Empirical evidence suggests that well-implemented street greenery initiatives significantly boost the duration and frequency with which residents engage in walking and cycling (15–18). The effectiveness of street greenery in promoting active travel is likely rooted in its capacity to enhance the visual appeal of urban environments, offering shade and cooler temperatures, which collectively contribute to increased comfort for pedestrians and cyclists (19, 20). This phenomenon can be understood through three key intermediary mechanisms. Firstly, street greenery contributes to creating an esthetically pleasing and comfortable environment, which has been shown to influence route choice and walking behavior positively. This is supported by research that highlights the importance of well-designed and high-quality community structures in encouraging active travel (21). By enhancing pedestrian pathways and beautifying community spaces, street greenery renders these areas more attractive, thereby fostering environments conducive to recreational and active travel behaviors. Secondly, the improvement of air quality and reduction of noise levels play a crucial role in facilitating active travel. A range of studies has demonstrated the adverse effects of subjective noise perception and PM2.5 exposure on individuals’ satisfaction with their travel experiences (22–24). A specific study elucidates how exposure to green streets can both directly and indirectly augment walking satisfaction among residents (25). It reveals that mitigating factors such as noise and PM2.5 levels are significant, underlining the direct positive influence of street greenery on walking satisfaction, as well as its indirect benefits through environmental enhancements. Improved air quality not only boosts energy levels and cognitive focus but also aids in mitigating the risk of neurological abnormalities (26). Moreover, a serene ambiance, achieved by reducing noise disturbances, provides a more enjoyable experience for active travelers, thereby encouraging them to incorporate active modes of transportation into their daily routines (27). Furthermore, street vegetation acts as a natural buffer against air and noise pollution, thereby creating an inviting and conducive environment for active travel (25). Finally, street greenery supports social interactions and fosters a sense of community, offering residents additional reasons to opt for walking, biking, and other active travel modes (28). Understanding these mediating mechanisms is crucial in discerning the multifaceted ways through which street greenery can influence active travel. By improving the esthetic appeal, environmental quality, and social cohesiveness of urban communities, street greenery initiatives can significantly promote sustainable active travel behaviors.

Nonetheless, the precise nature of the relationship between street greenery and active travel remains elusive, with the current body of research presenting a disjointed picture. Some studies assert a strong positive association (15, 29), while others report negligible or no correlation (30–32). These discrepancies could stem from methodological divergences, varying metrics for evaluating street greenery, or differing population demographics.

This review systematically examines the impact of street greenery on active travel. Our aim is threefold: First, we synthesize existing research to identify patterns and differences in findings, thereby elucidating the relationship between street greenery and active travel. Second, we critically analyze these studies to identify gaps and methodological limitations, setting the stage for future detailed investigations. Lastly, we aim to provide urban planners and policymakers with concrete insights about the role of street greenery in promoting pedestrian and cycling activities. This will assist in leveraging green infrastructure for active transportation, ultimately contributing to public health, environmental sustainability, and urban space enhancement. The primary purpose of this study, therefore, is to provide a comprehensive understanding of how street greenery influences active travel and to inform the development of effective urban planning strategies.

## 2 Methods

The current research adhered to the guidelines set by the Preferred Reporting Items for Systematic Reviews and Meta-Analyses (33).

### 2.1 Study selection criteria

This systematic review included studies based on a comprehensive set of inclusion criteria: (1) study design: experimental design, cross-sectional studies; (2) participants: individuals from all age groups; (3) exposure variable: street greenness, street vegetation such as trees, shrubs and lawns; (4) outcome measures: active travel behaviors, such as walking, cycling; (5) article type: peer-reviewed articles; (6) retrieval time window: from the inception of the relevant electronic bibliographic database until 21 June 2023; (7) geographical scope: global scale; (8) language: articles written in English.

The exclusion criteria were as follows: (1) studies that did not directly address active travel behaviors, such as walking or cycling; (2) studies did not involve street greenness; (3) studies published in a language other than English, to maintain linguistic consistency and facilitate uniform analysis; (4) the document type was a letter, editorial, research or review proposal, or a review article, as these types of documents typically do not provide original empirical findings.

### 2.2 Search strategy

A search for relevant keywords was executed across four major electronic bibliographic databases, namely PubMed, Web of Science, Scopus, and Cochrane Library. The search strategy encompassed all potential permutations of keywords associated with the three specified categories: (1) “street,” “eye-level,” “streetscape,” “street-level,” “street-side”; (2) “greenspace,” “greenspaces,” “green-space,” “green space,” “green spaces,” “green infrastructure,” “green infrastructures,” “green area,” “green areas,” “green belt,” “green belts,” “green environment,” “green environments,” “greening project,” “green element,” “green elements,” “urban green,” “greenery,” “greenness,” “greenbelt,” “greener,” “natural element,” “natural elements,” “natural environment,” “natural environments,” “natural outdoor environment,” “natural outdoor environments,” “natural surroundings,” “natural space,” “natural spaces,” “natural area,” “natural areas,” “natural land,” “open space,” “open spaces,” “open land,” “open area,” “open areas,” “walkable area,” “walkable areas,” “vegetated area,” “vegetated areas,” “public space,” “public spaces,” “public area,” “public areas,” “public land,” “nature,” “vegetation,” “park,” “parks,” “parkland,” “garden,” “gardens,” “tree,” “trees,” “landscape,” “woodland,” “woodlands,” “walkability”; (3) “active travel,” “bike,” “biking,” “bicycle,” “bicycling,” “cycling,” “active school transport,” “active transport,” “active transportation,” “active transit,” “active commuting,” “travel mode.” In the PubMed database, we utilized the “[TIAB]” tag to execute a thorough keyword search, ensuring that the title and abstract of the articles were meticulously combed for relevant terms. For the Web of Science database, we engaged the TS = Topic search tool, which extends the search through the article’s title, abstract, keywords, and Keywords Plus fields, providing a broad sweep across diverse academic disciplines. This search strategy set the stage for an intensive initial review phase. During this phase, the articles retrieved via keywords underwent a detailed evaluation against our stringent study selection criteria, based on their titles and abstracts. Those articles exhibiting preliminary signs of relevance were advanced to the next level for an in-depth full-text review. To maintain objectivity and thoroughness, this preliminary filtering process was independently conducted by two of the co-authors involved in this review. The concordance rate between the reviewers was quantified using Cohen’s kappa statistic, which yielded a substantial agreement score of κ = 0.77. Any discrepancies were resolved by consulting a third co-author.

To ensure the exhaustive coverage of literature, we conducted a meticulous backward and forward reference search, examining the reference lists of the selected full-text articles and tracking their citation trails. This bidirectional search allowed us to identify and incorporate studies that may not have been captured through keyword searches alone. Each article surfaced from this recursive search underwent a stringent screening using the same selection criteria established for the initial review. This iterative process was repeated until saturation was reached, with no further pertinent articles emerging.

### 2.3 Data extraction and preparation

Data from each article was meticulously collated using a uniform data extraction template. The table facilitated the systematic collection of vital information, including the author(s)’ names, year of publication, country of study, design methodology, sample size, participant age range, proportion of female participants, sample characteristics, statistical model, control variables, type of street greenness measure, detailed measure of street greenness, type of active travel measure, and detailed measure of active travel.

### 2.4 Data synthesis

The data compilation for this review was meticulously orchestrated by two co-authors. Our report encapsulates a detailed synthesis of the prominent themes and insights derived from the analyzed studies. The methodical procedures of data acquisition, thematic delineation, and synthesis were independently executed by two co-authors. Encountered discrepancies were diligently reconciled through a consultative discourse involving a third co-author, thereby upholding the analytical coherence of our review.

### 2.5 Study quality assessment

We appraised the methodological soundness of each study using the National Institutes of Health’s Observational Cohort and Cross-sectional Study Quality Assessment Tool, which assesses studies against a set of 14 criteria. For each criterion met, a study was awarded a point (“yes”), with no points given for unmet criteria (“no,” “not applicable,” “not reported,” or “indeterminate”). The cumulative points for all criteria yield a study’s overall quality score, which ranges from 0 to 14. This quality assessment, while crucial for evaluating the strength of the evidence presented, did not influence the decision to include studies in our review. Discrepancies in the quality assessment conducted by two co-authors were resolved through a consultative process with a third co-author, ensuring an unbiased and consistent evaluation.

## 3 Results

### 3.1 Identification of studies

**Figure 1:**
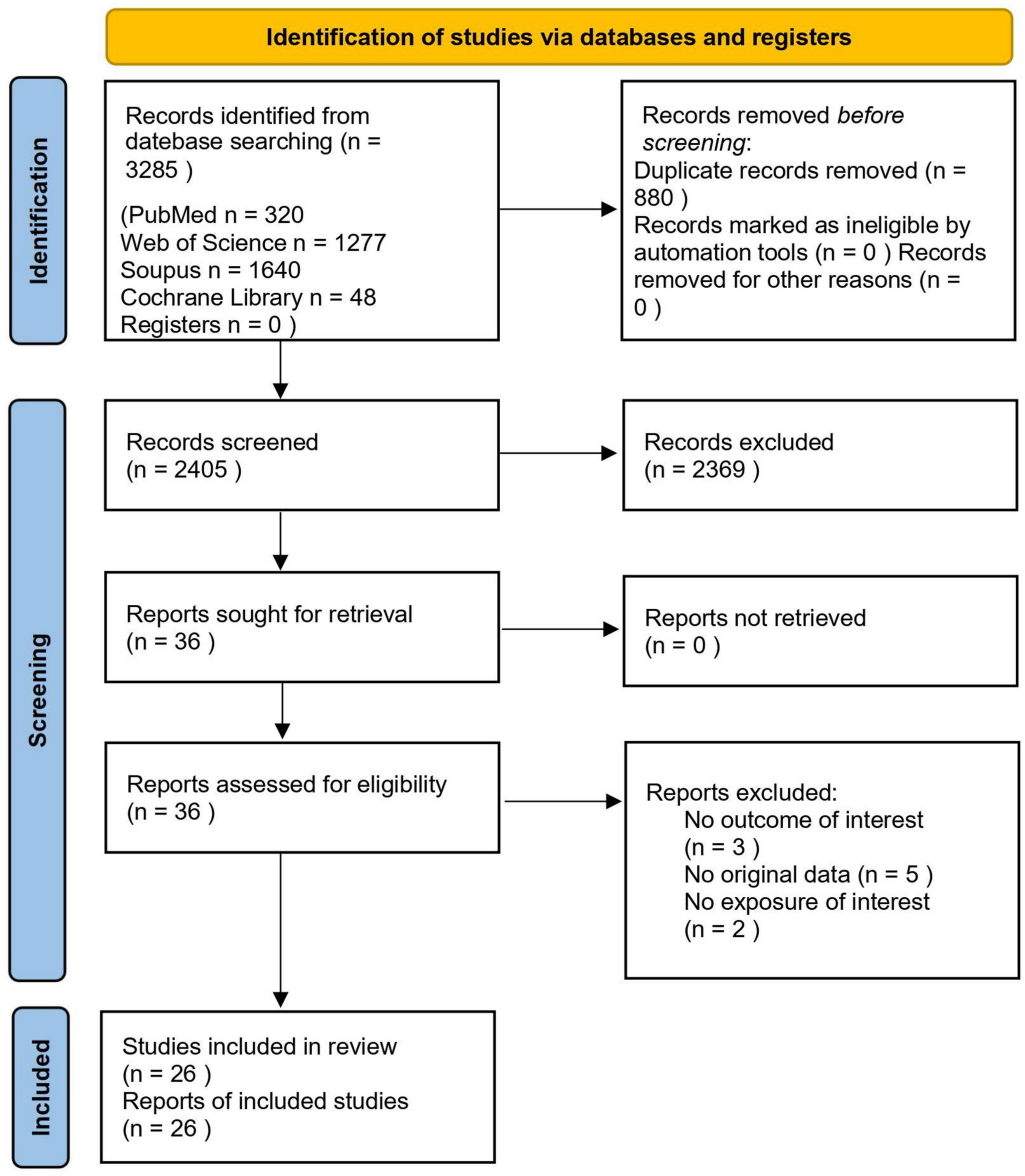
Study selection flowchart.

Figure 1 illustrates the study selection process. A total of 3,285 articles were initially identified through keyword searches and reference screening. After removal of duplicates (880 articles), the remaining 2,405 articles underwent title and abstract screening, resulting in the exclusion of 2,369 articles. Subsequently, a full-text review was conducted on the remaining 36 articles in accordance with the study selection criteria. Among these, 10 articles were excluded. The primary reasons for exclusion included a lack of street greenery data in the articles, absence of reported outcomes regarding active travel. As a result, the final analysis comprised 26 studies that investigated the influence of street greenery on active travel (13, 15–18, 25, 29–32, 34–49).

### 3.2 Basic characteristics of the included studies

Table 1 summarizes the fundamental characteristics of the literature incorporated in this study. All studies adopted a cross-sectional study design. In total, this review encompasses 26 studies, with 19 of them originating from China. Among these, 11 were conducted in mainland China, while the remaining 8 were carried out in Hong Kong, China. Additionally, 3 studies were conducted in the United States, with 1 study each conducted in the United Kingdom, Spain, the Netherlands, and Korea. All the included studies were published in 2015 or later, with 1 study published in 2015, 4 studies in 2018, 2019, 2020, 2022, and 2023, and 5 studies in 2021. Among the 26 studies included in the analysis, there is considerable variation in sample sizes. Eight studies had sample sizes ranging from 127 to 999 participants. Six studies had sample sizes between 1,000 and 9,999 participants, while five studies had sample sizes exceeding 10,000 participants. Five studies reported trip records as their sample size, ranging from nearly 140,000 to 20 million records. The remaining two studies did not report their sample sizes. Among these studies, one focused on a university student population, five concentrated on adults, and four specifically studied the older adults. Residents from various age groups, while seven studies did not report the characteristics of the study sample. With the exception of eight studies that did not report gender ratios, all studies included both male and female participants, with a generally balanced gender distribution. These studies applied a variety of statistical models, including logistic regression, continuous regression, spatial error regression, multilevel logistic regression, multilevel linear regression, geographically weighted regression, binary logistic regression, ordinary least square models, structural equation models, and multivariate Poisson regression models. Most studies utilized individual socio-demographic information, such as age, gender, occupation, household income, as control variables. Some studies also incorporated control variables such as population density, street intersection density, land use mix, and others.

**Table 1:** Basic characteristics of the studies included in the review.

Study ID	First author (year)	Country	Study design	Sample size	Age (years)	Female (%)	Sample characteristics	Statistical model	Control variables
1	Sarkar, 2015 (34)	UK	Cross-sectional	15,354 respondents	39.4 ± 20.9	52.2	Residents of all ages	Logistic regression, continuous regression	Age, gender, ethnic group, prevalence of disability, access to vehicles and household income, urban morphology and accessibility, neighborhood-level deprivation and road safety
2	Li, 2018 (35)	USA	Cross-sectional	300,000 trips	NA	NA	NA	Spatial error regression	Spatial autocorrelation, walk score, population
3	Lu, 2018a (36)	China	Cross-sectional	The odds of walking: 24773 residents; total walking time: 1994 residents	5+	51.9, 56.6	Residents of all ages	Logistic regression, linear regression	Age, gender, household income, other built environment covariates
4	Lu, 2018b (15)	China	Cross-sectional	The odds of walking: 90,445 participants; total walking time: 6770 participants	2+	53.04, 57.37	Residents	Multilevel logistic regression, multilevel linear regression	Age, gender, income, vehicle ownership, urban density, street connectivity, land-use mix, number of bus stops and retail stores, and distance to the closest Mass Transit Rail station
5	Lu, 2018c (37)	China	Cross-sectional	1,390 participants	53 ± 20	51	Residents of all ages	Multilevel logistic regression	Household income, gender, age, population density, street intersection density, land use mix, and total park area
6	Lu, 2019 (38)	China	Cross-sectional	5,701 participants	15+	51.5	Residents	Multilevel logistic regression models	Gender, age, household income, population density, street intersection density, land-use mix, cycling lane density, number of bus stops and retail stores, terrain slope, and distance to the closest Mass Transit Rail station
7	Tsai, 2019 (30)	US	Cross-sectional	423 residents	NA	60	Residents of all age	Linear regression, logistic regression	City of residence, residential length, survey season, sex, age, race/ethnicity, employment status, education attainment, economic hardship index, perception of community safety from crime, perceived walking to multiple destinations within 10 min, and estimated intersection density of walkable roads/land use mix
8	Vich, 2019 (39)	Spain	Cross-sectional	127 participants	18–64	59.1	Adults	Multilevel logistic regression	Gender, age and commuting mode, public and private transport
9	Yang, 2019 (40)	China	Cross-sectional	The odds of walking: 10700 participants; total walking time: 1083 participants	65–80	50.5	Older adults	Multilevel logistic regression	Age, gender, household, vehicle
10	Chen, 2020 (29)	China	Cross-sectional	901,760 records of shared bicycles usage	NA	NA	NA	Linear regression	NA
11	Wang, 2020 (16)	China	Cross-sectional	20 million cycling trips	NA	NA	NA	Multilevel logistic regression	Population density, street intersection density, land-use mix, the number of bus stops, retail stores, terrain slope.
12	Wu, 2020 (31)	China	Cross-sectional	791 participants	16–64	55.5	Residents	Multi-level logit regression and multi-level linear regression	Gender, age, hukou, education, marriage, income, license, employment, car number, work time, working and living distance, day type, travel time flexibility, vehicle flexibility, starting place, ending place
13	Zang, 2020 (41)	China	Cross-sectional	180 participants	65–85	35	Older adults	Logistic regression	Age, gender, former occupation, self-reported health status
14	Gao, 2021 (42)	China	Cross-sectional	NA	NA	NA	NA	Multi-level linear regression, geographically weighted regression	Normalized difference vegetation index, floor area ratio, land use entropy, real-time, population density
15	Ki, 2021 (18)	Korea	Cross-sectional	2,500 residents	20–65	60	Residents	Logistic regression	Sex, age, self-selection, household income, housing type, walking time by purpose
16	Ta, 2021 (43)	China	Cross-sectional	791 respondents	18+	NA	Working-age residents	Logistic regression	Active travel, trip duration, trip purpose, departure time, peak time, weekend, last activity satisfaction before the trip, PM2.5 level, average green exposure on daily trips, female, marriage, age, education, house ownership, income
17	Yang, 2021a (44)	China	Cross-sectional	10,700 participants	73.82	51	Residents	Logistic regression	House type, male, automobile, income
18	Yang, 2021b (13)	China	Cross-sectional	1,083 participants	65+	53	Residents	Logistic regression, linear regression	Family size, male, age, family income, population density, land use mix, intersection density, access to the metro, access to recreational facilities
19	Bai, 2022 (45)	China	Cross-sectional	965 participants	22 ± 5.2	61	University students	Multilevel logistic regression model	Gender, age, educational attainment, income, travel tools ownership, hukou status, travel satisfaction
20	Koo, 2022 (46)	USA	Cross-sectional	318 participants	42.7 ± 17.0	42.1	Residents	Binary logistic regressions	Age, gender, race, educational attainment, number of vehicles owned by the household, household income, driver status, number of walking activities in the past 7 days, travel distance of each trip
21	Luo, 2022 (32)	China	Cross-sectional	NA	NA	NA	NA	Ordinary least square model, geographically weighted regression	Far, land use mix, river line length, road density, green space area, number of bus stations, number of enterprises
22	Song, 2022 (25)	China	Cross-sectional	144 participants	15–60	52.78	Residents	Structural equation model	Walking companions, walking duration, bus stops density, retail store density, socio-demographic attributes, family-related attributes
23	Bai, 2023 (47)	China	Cross-sectional	139,018 trips	NA	NA	NA	Multivariate Poisson regression model	Normalized difference vegetation index, land-use mix, greenway link-node ratio, building density, number of parks and plazas, greenway width.
24	Gao, 2023 (48)	China	Cross-sectional	2,495,848 trips	NA	NA	NA	Poisson regression	Population density, building floor area ratio, road density, land use mix entropy, distance to subway station, distance to bus station, distance to city center
25	Liu, 2023 (49)	Netherlands	Cross-sectional	1886 participants	18+	58	Residents	Generalized additive mixed models	Gender, nationality, possession of a driving license, bicycle ownership, education level, monthly household income, household car ownership
26	Xie, 2023 (17)	China	Cross-sectional	1,020 participants	50.8 ± 16	56.6	Residents	Linear regression, logistic regression	Gender, age, employment status, marital status, educational attainment, annual household income, homeownership, self-rated health status, household member, travel mode to the greenway

NA, not applicable.

Table 2 provides a comprehensive overview of the measurement methods used in the included studies for assessing street greenness and active travel. It also highlights the specific variables related to these street greenness and active travel. Across all 26 studies, objective measurement were employed to assess street greenness. In particular, 10 studies used data sourced from Google Street View images, nine studies relied on Baidu street view maps, two studies utilized Tencent street view Map, and the remaining five studies employed various other objective measurement, including data from EnviroAtlas, GPS tracking points obtained through the MOVES smartphone app, and information accessed from Amsterdam’s data portal. The specific indicators used to assess street greenness primarily focused on eye-level street greenness, evaluated using the Green View Index (GVI). Other indicators encompassed metrics such as street tree density, street tree cover, sidewalk tree cover, and greenway proximity. Regarding active travel, it primarily encompassed walking (*n* = 13), cycling (*n* = 6), and active transportation (*n* = 7). Data related to active travel were drawn from a variety of sources, including large-scale survey like the London Travel Demand survey (*n* = 1), the Hong Kong Travel Characteristics Survey (*n* = 5), survey of the Health of Wisconsin (*n* = 1), the National Household Travel Survey (*n* = 1), and the Dutch National Travel Survey (*n* = 1); self-reported questionnaire (*n* = 6), with two of them using the International Physical Activity Questionnaire; objective measurement (*n* = 8), with two utilizing smartphone app, five obtained from bike-sharing companies, and one obtained from Strava; Additionally, the remaining two studies adopted a combined approach, incorporating both objective data obtained from GPS tracking and subjective travel diaries.

**Table 2:** Measures of street greenness and active travel in the studies included in the review.

Study ID	First author (year)	Type of street greenness measure	Detailed measure of street greenness	Type of active travel measure	Detailed measure of active travel
1	Sarkar, 2015 (34)	Objective measure	The density of street trees	Self-reported questionnaire: London Travel Demand survey	Walking distance
2	Li, 2018 (35)	Objective measure: derived from Google Street View images	The amount of street greenery: Green View Index	Objective measure: collected from a smartphone application	Walking activities: trip number
3	Lu, 2018a (36)	Objective measure: derived from Google Street View images	The eye-level street greenness	Objective measure	The odds of walking Total walking time
4	Lu, 2018b (15)	Objective measure: derived from Google Street View images	The availability of eye-level street greenery	Self-report questionnaire: HKTCS	The odds of walking Total walking time
5	Lu, 2018c (37)	Objective measure: derived from Google Street View images	The quantity and quality of street greenery	Self-reported questionnaire: IPAQ	The total duration of green physical activity (≥150 min/week vs. 150 min/week)
6	Lu, 2019 (38)	Objective measure: derived from Google Street View images	Eye-level street greenness	Self-reported questionnaire: HKTCS	The odds of cycling
7	Tsai, 2019 (30)	Objective measure: EnviroAtlas	Street tree cover	Self-report questionnaire: survey of the Health of Wisconsin	The odds of walking or cycling Frequency of walking or cycling Duration of walking or cycling
8	Vich, 2019 (39)	Objective measure: GPS tracking points obtained from MOVES smartphone app	Environmental exposure to greenness	Objective measure: GPS tracking points obtained from MOVES smartphone app	Walking patterns: distances, durations, steps, and burned calories
9	Yang, 2019 (40)	Objective measure: derived from Google Street View images	The level of street greenery	Self-report questionnaire: HKTCS	The odds of engaging in walking Total walking time
10	Chen, 2020 (29)	Objective measure: derived from Tencent Street View	Green view index	Objective measure: captured the data from Mobike	The use of dockless shared bicycles
11	Wang, 2020 (16)	Objective measure: derived from Tencent Online Map	Eye-level street view greenness	Objective measure: obtained from bike-sharing company Mobike	Cycling frequency
12	Wu, 2020 (31)	Objective measure: derived from Baidu Maps street view image	Street green view index	Objective measure: obtained from GPS tracking Self-report questionnaire: obtained from the travel diary	The probability of Active Travel Active Travel distance, duration
13	Zang, 2020 (41)	Objective measure: obtained from Baidu Street View images	Street green view index	Self-report questionnaire: IPAQ	Total walking time
14	Gao, 2021 (42)	Objective measure: obtained via BaiduMap street view images	Eye-level urban greenness	Objective measure: obtained from the bike sharing operators (Mobike, Ofo, Bluegogo, and Xiaoming Bike)	Bike sharing usage
15	Ki, 2021 (18)	Objective measure: derived from Google Street View images	Street green view index	Self-report questionnaire	Walking time
16	Ta, 2021 (43)	Objective measure: obtained from Baidu Maps Street View images	Street Green View Index	Objective measure: obtained from GPS tracking devices Self-report questionnaire: obtained from the daily activity diary	Active travel satisfaction
17	Yang, 2021a (44)	Objective measure: derived from Google Street View images	Eye-level streetscape greenery	Self-report questionnaire: HKTCS	Walking propensity
18	Yang, 2021b (13)	Objective measure: Google Street View imagery	Eye-level street greenery index	Self-report questionnaire: HKTCS	Walking time
19	Bai, 2022 (45)	Objective measure: derived from Baidu Maps street view images	Street greenery	Self-report questionnaire	Active travel preference
20	Koo, 2022 (46)	Objective measure: derived from Google Street View images	Streetscape factors: greenness	Self-report questionnaire: National Household Travel Survey	The odds of walking
21	Luo, 2022 (32)	Objective measure: derived from Baidu Maps Street View images	Street green view index	Objective measure: Strava data	Cycling and running activities
22	Song, 2022 (25)	Objective measure	Eye-level street greenness	Self-report questionnaire: obtained from the daily activity diary	Walking satisfaction
23	Bai, 2023 (47)	Objective measure: street-view images from Baidu Maps	Street-view greenness	Objective measure: obtained from the daily order dataset of bicycle-sharing companies updated by the Shenzhen government’s open data platform	Cycling frequency
24	Gao, 2023 (48)	Objective measure: derived from Baidu Maps street view images	The eye-level street greenery view index	Objective measure: obtained from Mobike	Bike sharing usage
25	Liu, 2023 (49)	Objective measure: obtained geo-tagged street view images from Amsterdam’s data portal	Street greenery	Objective measure: obtained from Dutch National Travel Survey	Walking duration
26	Xie, 2023 (17)	Objective measure: using the street network analysis provided by Baidu Maps	The greenway proximity	Self-report questionnaire	The use of the East Lake Greenway: frequency, time, and intensity

HKTCS, Hong Kong Travel Characteristics Survey; IPAQ, International Physical Activity Questionnaire.

### 3.3 Key findings

Table 3 presents the key findings derived from the included studies, elucidating the intricate relationship between street greenness and active travel behavior. We provide a concise summary of how street greenness influences various facets of active travel, encompassing factors like active travel duration or distance, the probability of active travel engagement, active travel frequency, and active travel satisfaction.

**Table 3:** Estimated effects of street greenness on active travel in the studies included in the review.

Study ID	First author (year)	Estimated effects of street greenness on active travel	Main findings of this study
1	Sarkar, 2015 (34)	1. The regularity observed was that a higher density of street trees consistently correlated with an increased likelihood of walking (OR = 1.06, 95% CI = 1.03–1.10). 2. Among the factors related to street-level design and accessibility, local-scale betweenness at a 400-meter radius was found to have a positive association with walking. Compared to the lowest quartile, the second and fourth quartiles exhibited significantly increased odds of walking (1.15, 95% CI = 0.99–1.32 and 1.29, 95% CI = 1.09–1.53, respectively in model 3). 3. Conversely, when examining meso-scale betweenness at a 3,000-meter radius, it was linked to a reduced likelihood of falling into the category of individuals who engage in some walking (with odds ratios of 0.86, 95% CI = 0.75–0.99; 0.84, 95% CI = 0.72–0.98; and 0.84, 95% CI = 0.71–0.99 for the second, third, and fourth quartiles, respectively). 4. The density of street trees exhibited a consistent positive correlation with the distance walked, which remained stable across all models after accounting for other factors (0.056, 0.025–0.088 for model 1; 0.055, 0.024–0.086 for model 2; 0.039, 0.007–0.071 for model 3). 5. The association between street-level betweenness and the distance walked remains statistically non-significant.	1. There is a notable correlation between the odds of walking and the density of street trees as well as street-level betweenness (which measures street network connectivity). 2. The sensitivity analyses involving continuous regression models for individuals engaged in some walking revealed favorable associations between the distance walked and street trees.
2	Li, 2018 (35)	1. In the cases of residential land, commercial land, recreational land, and industrial land, there is no statistically significant association between the street enclosure by trees and the trip number.	1. The relationship between the visibility of street greenery and human walking activities varies depending on the land use types. 2. There is no statistically significant association between street enclosure by trees and human walking activities in any of the land use types.
		2. In the case of residential and commercial land, there is a notable and negative correlation between the green view index and the trip number (with coefficients of −1.50 × 10–2, *p* < 0.01 for residential land and − 1.35 × 10–2, *p* < 0.01 for commercial land). However, for recreational and industrial land, there is no significant association between the green view index and the trip number.	
3	Lu, 2018a (36)	Green view index and the odds of walking:	Eye-level greenery was found to be significantly associated with increased odds of walking and extended walking time within both the 400-meter and 800-meter buffers.
		400 m Buffer: OR = 1.149, 95%CI = 1.035, 1.276, *p* = 0.009	
		800 m Buffer: OR = 1.193, 95%CI = 1.070, 1.330, *p* = 0.001	
		Green view index and walking time:	
		400 m Buffer: β = 0.149, 95%CI = 0.045, 0.253, *p* = 0.005	
		800 m Buffer: β = 0.223, 95%CI = 0.133, 0.333, *p* < 0.001	
4	Lu, 2018b (15)	Street greenery and the odds of walking (800 m Buffer):	1. Street greenery was associated with higher odds of walking.
		Level of street greenery (middle-high vs. low): OR = 1.07, 95%CI = 1.01, 1.13, *p* = 0.023	
		Level of street greenery (high vs. low): OR = 1.09, 95%CI = 1.02, 1.16, *p* = 0.009	2. Street greenery was linked to the total time spent walking.
		Street greenery and walking time (800 m Buffer):	
		β = 0.09, 95%CI = 0.04, 0.14, *p* < 0.001.	
5	Lu, 2018c (37)	Street greenery and achieving ≥150 min of recreational green physical activity a week (1,000 m Buffer):	The presence and amount of street greenery showed a positive correlation with recreational physical activity.
		Quantity of street greenery (high vs. low): OR = 1.20, 95%CI = 1.08, 1.33, *p* = 0.02	
		Quality of street greenery (high vs. low): OR = 1.10, 95%CI = 1.05, 1.25, *p* < 0.01	
6	Lu, 2019 (38)	Street greenness and the odds of cycling:	There was a positive correlation between the likelihood of cycling and eye-level street greenness within three different buffer zones: 400 m, 800 m, and 1,600 m.
		400 m buffer: OR = 1.21, 95%CI = 1.00, 1.46;	
		800 m buffer: OR = 1.25, 95%CI = 1.04, 1.51;	
		1,600 m buffer: OR = 1.36, 95%CI = 1.11, 1.67.	
7	Tsai, 2019 (30)	A 10% increase in sidewalk tree cover and odds of participating in active transportation:	1. The probability of engaging in active transportation was positively linked sidewalk tree cover within all network buffers. 2. Street tree cover did not demonstrate a significant association with active transportation at a 500 m radius in any of the models. However, within network buffers ranging from 750 m to 1,250 m, street tree cover exhibited a positive correlation with active transportation.
		500 m buffer: AOR: 1.19; 95% CI: 1.02–1.40 (*p* < 0.05);	
		750 m buffer: AOR: 1.25; 95% CI: 1.05–1.49 (*p* < 0.05);	
		1,000 m buffer: AOR: 1.27; 95% CI: 1.05–1.54 (*p* < 0.05);	
		1,250 m buffer: AOR: 1.31; 95% CI: 1.08–1.61 (*p* < 0.01).	
		A 10% increase in street tree cover and odds of participating in active transportation:	
		500 m buffer: AOR: 1.19; 95% CI: 0.99–1.43 (*p* > 0.05);	
		750 m buffer: AOR: 1.27; 95% CI: 1.03–1.57 (*p* < 0.05);	
		1,000 m buffer: AOR: 1.32; 95% CI: 1.05–1.67 (*p* < 0.05);	
		1,250 m buffer: AOR: 1.39; 95% CI: 1.09–1.79 (*p* < 0.01).	
8	Vich, 2019 (39)	Tree density and walking time:	The presence of street trees showed a positive correlation with individual walking activity levels.
		B = 0.001, SE = 0.000, t = 5.895, *p* = 0.001, 95%CI = 0.000, 0.000.	
9	Yang, 2019 (40)	Street greenery and the odds of walking (800 m buffer):	The presence of street greenery exhibited a positive association with both the likelihood of older adults engaging in walking and the total time they spent walking.
		OR = 1.165, 95%CI = 1.004, 1.352, *p* = 0.04.	
		Street greenery and walking time:	
		β = 0.187, (95% CI = 0.071, 0.304, *p* = 0.002).	
10	Chen, 2020 (29)	Green view index and riding density:	Eye-level greenery had a beneficial effect on cycling.
		Workday: coefficient = 0.061, *p* < 0.001;	
		Weekend: coefficient = 0.049, *p* < 0.01;	
		Total: coefficient = 0.054, *p* < 0.001.	
11	Wang, 2020 (16)	Street-view greenness and cycling frequency on weekdays:	1. Eye-level greenery showed a positive correlation with the frequency of cycling on both weekdays and weekends within three different buffer sizes around metro stations (500-m, 1,000-m, and 1,500-m). 2. The impact of eye-level greenery on cycling frequency was more pronounced during weekends compared to weekdays.
		500 m buffer: coefficient = 1.983, SE = 0.026, *p* < 0.01;	
		1,000 m buffer: coefficient = 2.095, SE = 0.023, *p* < 0.01;	
		1,500 m buffer: coefficient = 2.551, SE = 0.028, *p* < 0.01.	
		Street-view greenness and cycling frequency on weekends:	
		500 m buffer: coefficient = 2.520, SE = 0.027, *p* < 0.01;	
		1,000 m buffer: coefficient = 2.728, SE = 0.024, *p* < 0.01;	
		1,500 m buffer: coefficient = 3.807, SE = 0.029, *p* < 0.01.	
12	Wu, 2020 (31)	Green View Index and the probability of Active Travel:	1. The greater the cumulative Green View Index value, the lower the likelihood of increasing the probability of active travel. 2. The average Green View Index significantly increases the incidence of active travel. 3. The buildup of the Green View Index is significantly and positively associated with the distance of active travel. 4. The average Green View Index has a significant adverse impact on both walking and bicycle travel distances.
		Total green view index: coefficient = −0.001, SE =0.000, *p* = 0.000;	
		Mean green view index: coefficient = 5.873, SE = 0.648, *p* = 0.000;	
		Green View Index and walking distance:	
		Total green view index: coefficient = 0.003, SE =0.000, *p* = 0.000;	
		Mean green view index: Coefficient = −1.513, SE = 0.215, *p* = 0.000;	
		Green View Index and cycling distance:	
		Total green view index: coefficient = 0.002, SE =0.000, *p* = 0.000;	
		Mean green view index: Coefficient = −2.195, SE = 0.374, *p* = 0.000.	
13	Zang, 2020 (41)	Green View Index and walking time (500 m buffer):	The Street Greenery View Index plays a role in enhancing the walking time of older adults.
		Coefficient = 0.137, *p* = 0.05.	
14	Gao, 2021 (42)	Street greenness and Bike use:	Eye-level greenery exhibited a positive correlation with the usage of bike sharing on weekdays, weekends, and holidays.
		On weekdays Coefficient = 4.57, *p* < 0.05	
		On weekend Coefficient = 3.96, *p* < 0.05	
		On holidays Coefficient = 4.01, *p* < 0.05	
15	Ki, 2021 (18)	Green View Index and utilitarian walking time:	A high Green View Index encourages both practical and recreational walking time.
		Coefficient = 11.070, t = 6.26, *p* < 0.01;	
		Green View Index and leisure walking time:	
		Coefficient = 4.241, t = 3.91, *p* < 0.01.	
16	Ta, 2021 (43)	Street-level green space exposure and active travel satisfaction:	1. Interacting with green spaces during travel enhances people’s overall travel satisfaction. 2. The impact of exposure to green spaces on travel satisfaction differs depending on the mode of travel, its duration, and its purpose. 3. Exposure to green spaces significantly influences satisfaction with walking, nonwork trips, and medium-duration trips.
		Coefficient = 0.91, *p* < 0.1;	
		Street-level green space exposure and walking satisfaction:	
		Coefficient = 1.23, *p* < 0.05;	
		Street-level green space exposure and travel satisfaction with nonwork trips:	
		Coefficient = 2.58, *p* < 0.05;	
		Street-level green space exposure and Traveling trips for more than 30 min:	
		Coefficient = 2.03, *p* < 0.1.	
17	Yang, 2021a (44)	Streetscape greenery and walking propensity:	Streetscape greenery positively influences the inclination for walking within a specific range, but beyond that range, the positive correlation dissipates.
		Coefficient = 3.316, *p* < 0.1	
18	Yang, 2021b (13)	Street greenery and the walking time:	1. Street greenery consistently and significantly impacts walking duration. 2. The impact of street greenery differs across different locations, with a notably greater effect observed in suburban areas. 3. The performance of various green view indices displays a high level of consistency.
		400 m buffer: coefficient = 32.949, t-stat = 2.48, *p* < 0.05;	
		800 m buffer: coefficient = 46.642, t-stat = 2.79, *p* < 0.01;	
		1,600 m buffer: Coefficient = 37.851, t-stat = 2.11, *p* < 0.05.	
19	Bai, 2022 (45)	Green Vegetation Index and the likelihood of respondents being willing to participate in AT:	1. The presence of street greenery on university campuses is linked to a positive correlation with active travel among university students. 2. Modes of transportation also played a role in influencing active travel among university students, with those who owned bicycles being more inclined to engage in active travel. Conversely, those who relied on electric bikes were less likely to participate in active travel.
		Green vegetation index (Moderate vs. low): OR = 3.674, 95%CI = 1.162, 11.616, *p* < 0.05;	
		Green vegetation index (High vs. low): OR = 3.863, 95%CI = 1.443, 10.340, *p* < 0.01.	
20	Koo, 2022 (46)	Streetscape greenness and odds of walking: OR = 2.070, z-value = 2.655, *p* < 0.01.	The streetscape greenness exhibited a statistically significant positive association with a higher odds of walking.
21	Luo, 2022 (32)	1. The green view index and cycling index: coefficient = 0.138, *p* < 0.01. 2. The green view index and running index: coefficient = 0.028, *p* < 0.1.	1. In general, the Green View Index hinders cycling activities. 2. The Green View Index exhibits a positive correlation with running physical activity in specific regions (Dufu Thatched Cottage and Wukuaishi).
22	Song, 2022 (25)	Street greenness exposure and walking satisfaction: coefficient = 0.084, *p* < 0.05.	1. Exposure to street greenness has a substantial direct impact on walking satisfaction, as well as a significant indirect influence on walking satisfaction through subjective environmental annoyances (such as noise and PM2.5-related annoyances), rather than being mediated by objective noise and PM2.5 exposures. 2. In addition to physical activity and social interaction, it’s important to consider the indirect impact of street greenness exposure on walking satisfaction through subjective environmental pollution annoyance, which constitutes approximately 17.39% of the total effect and should not be overlooked.
23	Bai, 2023 (47)	Street-view greenness and cycling frequency:	Street-level greenery and the extent of greenway enclosure displayed a positive correlation with an increased frequency of cycling on both weekdays and weekends. However, the level of openness of the greenway seems to have contrasting effects on cycling frequency depending on the day of the week, as high levels of openness may promote cycling on weekends but impede it on weekdays.
		At Weekend: coefficient = 0.015, SE = 0.001, *p* < 0.01;	
		On Weekdays: coefficient = 0.014, SE = 0.001, *p* < 0.01;	
		In a Week: coefficient = 0.015, SE = 0.001, *p* < 0.01;	
		Street-view openness and cycling frequency:	
		At Weekend: coefficient = 0.005, SE = 0.001, *p* < 0.01;	
		On Weekdays: coefficient = −0.011, SE = 0.001, *p* < 0.01;	
		In a Week: coefficient = −0.003, SE = 0.001, *p* < 0.05;	
		Street-view enclosure and cycling frequency:	
		At Weekend: coefficient = 0.016, SE = 0.001, *p* < 0.01;	
		On Weekdays: coefficient = 0.016, SE = 0.001, *p* < 0.01;	
		In a Week: coefficient = 0.017, SE = 0.001, *p* < 0.01.	
24	Gao, 2023 (48)	Greenery view index and bike-sharing usage:	The Greenery View Index positively influences the usage of bike-sharing.
		All: incidence rate ratios = 1.003, z-value = 39.18, *p* < 0.001;	
		Weekday: incidence rate ratios = 1.003, z-value = 36.63, *p* < 0.001;	
		Weekend: incidence rate ratios = 1.003, z-value = 14.62, *p* < 0.001;	
		Morning: incidence rate ratios = 1.001, z-value = 5.695, *p* < 0.001;	
		Noon: incidence rate ratios = 1.007, z-value = 24.27, *p* < 0.001;	
		Evening: incidence rate ratios = 1.003, z-value = 12.98, *p* < 0.001.	
25	Liu, 2023 (49)	Street greenery and walking duration:	During weekends, there was a statistically significant positive association between street greenery and the duration of walking.
		On weekdays: coefficient = −0.006, SE = 0.006, *p* > 0.1;	
		For weekends: coefficient = 0.03, SE = 0.01, *p* < 0.05.	
26	Xie, 2023 (17)	Greenway proximity and greenway use frequency:	1. The proximity to greenways exhibited a negative correlation with both the frequency and intensity of greenway use. 2. Having good greenway proximity did not demonstrate a statistically significant association with the amount of time spent using greenways.
		Coefficient = −0.12, 95%CI = −0.19, −0.05, *p* < 0.01;	
		Greenway proximity and greenway use time:	
		Coefficient = −0.02, 95%CI = −0.08, 0.04, *p* > 0.1; Greenway proximity and greenway use intensity:	
		OR = 0.73, 95%CI = 0.54, 0.98, *p* < 0.05.	

#### 3.3.1 Street greenness and active travel duration, distance

Eleven studies were conducted to investigate the influence of street greenness on active travel distance or time (13, 15, 17, 18, 31, 34, 36, 39–41, 49). The findings revealed a positive correlation between street tree density and both walking distance (34) and walking duration (39). Notably, eye-level greenness, as indicated by the GVI, exhibited a significant relationship with extended walking durations within various buffer zones, including 400 m (13, 36), 500 m (41), 800 m (13, 15, 36, 40), and 1,600 m (47), for both utilitarian and leisure walking (45), particularly during weekends (49). Moreover, the cumulative GVI demonstrated a significantly positive correlation with active travel distance (31). However, it should be noted that the impact of street greenness on walking duration varies depending on the measurement, features, or weekdays/weekends. For instance, the mean Green View Index was found to have a significant negative effect on walking and bicycle travel distance (43). Furthermore, factors such as good greenway proximity and street-level betweenness did not show a significant association with walking distance or greenway utilization time (17, 34). Additionally, on weekdays, street greenness was not significantly related to walking duration (49), The impact of street greenery exhibits spatial variability, with a notably more pronounced effect observed in suburban areas (13).

#### 3.3.2 Street greenness and the odds of active travel engagement

Nine studies have investigated the relationship between street greenness and the likelihood of active travel engagement. These studies have yielded significant findings, with five of them specifically examining the influence of street greenness on the probability of walking behavior (15, 34, 36, 40, 46), one focused on its impact on cycling behavior (38), and three shedding light on its effects on the likelihood of engaging in active transportation (16, 17, 45). The research results consistently demonstrate a positive association between various aspects of street greenness and active travel. Firstly, a higher density of street trees was found to be consistently linked to an increased likelihood of walking (34). Additionally, eye-level greenness or street greenery were significantly associated with a higher probability of walking, particularly within 150 m (46), 400 m (36), and 800 m buffers (15, 36, 40). In the context of cycling, the odds of cycling were positively correlated with eye-level street greenness across three buffer zones: 400 meters, 800 meters, and 1,600 m (38). Furthermore, the probability of participating in active transportation showed a positive relationship with sidewalk tree cover across various network buffers, including 500, 750, 1,000, and 1,250 m. Moreover, the mean GVI was found to significantly increase the likelihood of engaging in active travel (31). Interestingly, street tree cover exhibited a positive association with active transportation, particularly within network buffers spanning 750 to 1,250 m. However, it is worth noting that street tree cover did not show a significant association with active transportation within the 500-m buffer (30). Intriguingly, as the accumulated value of the GVI increased, it was inversely related to the probability of active travel engagement (31).

#### 3.3.3 Street greenness and the frequency of active travel

Three studies have delved into the impact of street greenness on cycling frequency or greenway utilization frequency (16, 17, 47). Among these investigations, eye-level greenness emerged as a key factor, demonstrating a remarkable effect on cycling behavior. Notably, the effect of eye-level greenness on cycling frequency was found to be more pronounced on weekends than on weekdays (16). Additionally, street-view greenness and the level of greenway enclosure were positively correlated with increased cycling frequency, regardless of whether it was a weekday or a weekend (47). However, an intriguing finding emerged regarding the openness of the greenway. This factor seemed to yield divergent effects on cycling frequency depending on the day of the week. While high levels of greenway openness appeared to promote cycling on weekends, they potentially hindered it on weekdays (47). In contrast, greenway proximity demonstrated a somewhat unexpected trend. Greater proximity to greenways was negatively associated with greenway utilization frequency, implying that the convenience of access did not necessarily translate into higher usage of greenways (17).

#### 3.3.4 Street greenness and active travel satisfaction

Two studies have undertaken an examination of the impact of street greenness on satisfaction related to active travel (25, 43). Notably, exposure to green spaces was discovered to wield a substantial influence on the walking satisfaction (43). Moreover, it was discerned that street greenness exposure not only carries a notable direct effect on the level of satisfaction associated with walking, but also yields a significant indirect effect on walking satisfaction through the mediation of physical activity, social interaction, and subjective environmental annoyances, including noise and PM2.5-related annoyances (25).

### 3.4 Study quality assessment

Table 4 presents the detailed and overall quality ratings derived from the study quality assessment. On average, the studies achieved a score of 8.19, with a range from 7 to 9. Each study rigorously formulated its research questions and objectives, clearly defined the study population, adjusted for crucial potential confounding variables that could impact the relationship between exposure and outcomes, and ensured a minimum participation rate of 50%. The attrition rate was uniformly recorded at 20% or less across all 26 studies. During the same time period, participants were recruited from populations that were comparable or similar, with strict adherence to a set of predefined inclusion and exclusion criteria that were applied consistently across studies. Most of the studies (*n* = 21) examined different levels of the exposure as related to the outcome. Worth noting is that none of the 26 studies assessed the exposure of interest before outcome measurement, provided a sample size justification, power description, or variance and effect estimates, maintained a blinded status concerning the exposure status of participants. The research methodologies employed by the studies featured in this comprehensive review predominantly adhered to a cross-sectional design, entailing a solitary assessment during the study period, thereby precluding the ability to discern any temporal association between exposure and outcomes.

**Table 4:** Study quality assessment.

Study ID	1	2	3	4	5	6	7	8	9	10	11	12	13	14	15	16	17	18	19	20	21	22	23	24	25	26
Criterion																										
1. Was the research question or objective in this paper clearly stated?	Y	Y	Y	Y	Y	Y	Y	Y	Y	Y	Y	Y	Y	Y	Y	Y	Y	Y	Y	Y	Y	Y	Y	Y	Y	Y
2. Was the study population clearly specified and defined?	Y	Y	Y	Y	Y	Y	Y	Y	Y	Y	Y	Y	Y	Y	Y	Y	Y	Y	Y	Y	Y	Y	Y	Y	Y	Y
3. Was the participation rate of eligible persons at least 50%?	Y	Y	Y	Y	Y	Y	Y	Y	Y	Y	Y	Y	Y	Y	Y	Y	Y	Y	Y	Y	Y	Y	Y	Y	Y	Y
4. Were all the subjects selected or recruited from the same or similar populations (including the same time period)? Were inclusion and exclusion criteria for being in the study pre-specified and applied uniformly to all participants?	Y	Y	Y	Y	Y	Y	Y	Y	Y	Y	Y	Y	Y	Y	Y	Y	Y	Y	Y	Y	Y	Y	Y	Y	Y	Y
5. Was a sample size justification, power description, or variance and effect estimates provided?	N	N	N	N	N	N	N	N	N	N	N	N	N	N	N	N	N	N	N	N	N	N	N	N	N	N
6. For the analyses in this paper, were the exposure(s) of interest measured prior to the outcome(s) being measured?	N	N	N	N	N	N	N	N	N	N	N	N	N	N	N	N	N	N	N	N	N	N	N	N	N	N
7. Was the timeframe sufficient so that one could reasonably expect to see an association between exposure and outcome if it existed?	N	N	N	N	N	N	N	N	N	N	N	N	N	N	N	N	N	N	N	N	N	N	N	N	N	N
8 For exposures that can vary in amount or level, did the study examine different levels of the exposure as related to the outcome (e.g., categories of exposure, or exposure measured as continuous variable)?	N	Y	Y	Y	N	Y	Y	Y	Y	Y	Y	Y	Y	Y	Y	Y	Y	Y	N	N	Y	Y	Y	N	Y	Y
9. Were the exposure measures (independent variables) clearly defined, valid, reliable, and implemented consistently across all study participants?	N	N	Y	Y	Y	Y	N	Y	Y	N	Y	Y	Y	N	Y	N	Y	Y	Y	N	N	Y	N	N	N	N
10. Was the exposure(s) assessed more than once over time?	N	N	N	N	N	N	N	N	N	N	N	N	N	N	N	N	N	N	N	N	N	N	N	N	N	N
11. Were the outcome measures (dependent variables) clearly defined, valid, reliable, and implemented consistently across all study participants?	Y	Y	Y	Y	N	Y	N	Y	Y	Y	Y	Y	Y	Y	Y	Y	Y	Y	Y	Y	N	Y	Y	Y	Y	N
12. Were the outcome assessors blinded to the exposure status of participants?	N	N	N	N	N	N	N	N	N	N	N	N	N	N	N	N	N	N	N	N	N	N	N	N	N	N
13. Was loss to follow-up after baseline 20% or less?	Y	Y	Y	Y	Y	Y	Y	Y	Y	Y	Y	Y	Y	Y	Y	Y	Y	Y	Y	Y	Y	Y	Y	Y	Y	Y
14. Were key potential confounding variables measured and adjusted statistically for their impact on the relationship between exposure(s) and outcome(s)?	Y	Y	Y	Y	Y	Y	Y	Y	Y	Y	Y	Y	Y	Y	Y	Y	Y	Y	Y	Y	Y	Y	Y	Y	Y	Y
Total score	7	8	9	9	7	9	7	9	9	8	9	9	9	8	9	8	9	9	8	7	7	9	8	7	8	7

This study quality assessment tool was adopted from the National Institutes of Health’s Quality Assessment Tool for Observational Cohort and Cross-Sectional Studies. For each criterion, a score of one was assigned if “Y” was the response, whereas a score of zero was assigned otherwise. A study-specific global score, ranging from zero to 14, was calculated by summing up scores across all 14 criteria. Study quality assessment helped measure strength of scientific evidence, but was not used to determine the inclusion of studies.

## 4 Discussion

This systematic review comprehensively examines the impact of street greenery on active travel, drawing insights from 26 cross-sectional studies. The term “street greenery” refers to a variety of urban design features, such as street trees, planting strips, lawns, flower beds, pedestrian pathways, hedges, and green barriers. Active travel is defined to encompass walking and cycling behaviors, with data derived from both objective measures, such as mobile app data and bike-sharing, and subjective measures, including self-reported questionnaires. The findings indicate that a substantial proportion of the studies report a positive impact of street greenery on active travel. Nonetheless, the strength of this relationship appears to be modulated by various factors, including day of the week, age demographics, gender, the proximity of greenery, and the method of quantifying green spaces. Mechanistically, street greenery is posited to promote active travel through the creation of visually attractive, safe, and comfortable green environments, coupled with improvements in air quality, noise reduction, and facilitation of social engagement.

In our comprehensive review of 26 studies, 19 of them revealed a positive correlation between street greenery and various aspects of active travel, including the duration and distance of travel, participation probability, frequency, and satisfaction. These findings align with prior research, emphasizing the positive impact of green spaces on physical activity among Chinese adults (50) and associating street greenery with increased active commuting (51). However, inconsistencies surfaced across different variables, primarily attributed to variations in measurement methods. The choice of cumulative GVI and Mean GVI resulted in conflicting outcomes regarding the influence of street greenery on walking distance (31). Similar discrepancies arose in the probability of active travel participation, influenced by the measurement approach used (31). Additionally, disparities related to measurement periods, such as the positive correlation of eye-level greenness with weekend walking time but not on weekdays (49), and varying outcomes in different buffer zones (16, 36, 38, 49), underscore the nuanced impact of street greenery on active travel behavior. These inconsistencies may be attributed to regional disparities in geography, climate, culture, transportation infrastructure, and local travel habits.

Research highlights significant seasonal variations in residents’ active travel behaviors (30). Notably, active travel participation is substantially higher during non-winter months as compared to winter. This trend is attributed to the challenging conditions posed by colder temperatures and inclement weather in winter, which deter residents from engaging in active transportation (52). The seasonal dynamics of street greenery, which involves a range of plants, grasses, and trees, also play a pivotal role. In temperate climates, the visual appeal of street greenery changes with the seasons; spring and summer showcase lush vegetation and a high density of greenery, enhancing eye-level green visualization. Conversely, fall and winter see a reduction in this green vibrancy, as most plants, barring evergreens, shed their leaves and enter dormancy, leading to diminished eye-level greenness. This seasonal fluctuation in greenery may contribute to the observed seasonal differences in active travel, necessitating further research to understand its impact more comprehensively. Moreover, studies in environmental psychology have identified cultural and racial variations in leisure activities within green spaces (53–55), in the types of leisure activities engaged in the landscape. For example, one study (56) indicates differing patterns of park usage among American ethnic groups, with Hispanics typically engaging in more sedentary activities, while Whites often prefer walking or jogging, focusing on the esthetic aspects of parks. In contrast, Chinese residents frequently view parks as social gathering spaces, showing a preference for larger green areas with extensive recreational amenities and high-quality design (57). This suggests that cultural backgrounds significantly influence how green spaces are utilized, as further evidenced by a study (58) indicating that park usage in urban Hong Kong contrasts markedly with Western norms, possibly due to ethnic influences on recreational choices (59). Finally, the relationship between environmental amenities and active travel is also influenced by socio-economic factors. Globally, the distribution and management of urban green spaces are often inequitable (60, 61), with affluent neighborhoods typically having greater access to public parks and woody vegetation (62, 63). This disparity leaves lower-income, disadvantaged, and ethnic minority groups with limited green space access and minimal participation in urban forestry decision-making (61, 64). Consequently, in environmentally disadvantaged areas, low-income individuals are more inclined to opt for active travel for short distances, whereas high-income individuals in similar areas are less likely to do so (65).

The influence of street greenery on active travel is nuanced and varies across demographic factors such as age and gender. Studies targeting older adults and college students indicate divergent travel preferences, with older adults favoring quieter routes to destinations like supermarkets and restaurants (66, 67), while college students predominantly navigate within college campuses, relying on walking or bicycling due to limited public transportation options (45, 68). Notably, a gender disparity exists, with a lower percentage of older adult females engaging in cycling, attributed to perceived health limitations and security concerns (67, 69, 70). Understanding these demographic-specific factors is crucial for tailoring street greenery strategies to meet the diverse preferences of different age groups and genders. In addition to demographic factors, street greenery’s impact on active travel behavior is associated with economic income. Positive correlations are observed between street greenery distribution and housing prices, as well as street network density. Conversely, a negative correlation exists between street greenery and the proportion of socially vulnerable populations (71). Low-income individuals in environmentally disadvantaged areas are more inclined toward active transportation, while high-income individuals in environmentally affluent areas demonstrate a lower propensity for active travel (65), potentially linked to the spatial distribution of green spaces and higher rates of private vehicle ownership among wealthier households (72, 73). Furthermore, a negative association between household income and total walking time suggests that individuals with higher incomes exhibit a decreased likelihood of walking compared to their low-income counterparts (36, 40, 46, 49). Comprehensive evidence gathering is imperative to further explore how street greenery interacts with socioeconomic factors, influencing active travel behavior across various economic strata.

As highlighted in the 2021 Vienna Declaration and the Pan-European Master Plan on Cycling endorsed by the United Nations, active travel significantly impacts public health, necessitating innovative approaches to develop transportation and mobility systems that are clean, safe, healthy, and inclusive, aiming to reduce overreliance on the automobiles. This review provides valuable insights for urban planners, guiding street greenery initiatives. In the initial phases of urban street greenery planning, a thorough understanding of local demographics, including age, gender, and daily travel patterns, is essential. Categorizing roads based on their functions, such as accommodating traffic or serving recreational purpose, allows for the implementation of diverse buffer distances and specific street greenery features aligned with these needs. Policymakers and urban planners should concurrently prioritize enhancing the esthetics, comfort, quality, and safety of green spaces at eye level. Initiatives addressing air quality, road noise reduction, and design of multi-functional green spaces that foster social support are crucial. By creating an appealing environment, street greenery initiatives can enhance residents’ active travel experiences. Encouraging active travel behavior can be achieved through thoughtful and targeted urban planning strategies.

There are several limitations that warrant further improvement. Firstly, all included studies used an observational research design, posing challenges in establishing causal relationships. The lack of an experimental design prevents us from determining whether street greenery directly influences active travel or whether other factors may play a role. Therefore, future research could explore more experimental studies to better understand the causal relationship between street greenery and active travel. Secondly, the 26 studies included in this review were cross-sectional, limiting our observation to short-term changes in the relationship between street greenery and active travel. To comprehensively understand this relationship, it is essential to conduct longitudinal studies, providing additional insights into the dynamics over time. Furthermore, the exploration of intermediary mechanism through which street greenery affects active travel has remained predominantly theoretical, lacking empirical data analysis support. To address this gap, future research should focus on empirical data collection to delve into the intermediary mechanism. Conducting empirical studies will enable a more precise understanding of how street greenery influences active travel, offering valuable insights for urban planning and policymaking. By gathering empirical evidence, researchers can facilitate the development of supportive policies and strategies to create green street environments that are both friendly and comfortable, fostering active travel behavior.

## 5 Conclusion

This study provides a comprehensive and systematic review of the scientific evidence on the influence of street greenery on active travel, affirming its positive impact. However, the extent of this influence varies with factors such as temporal factors (weekdays vs. weekends), demographic segments (age and gender), proximity parameters (buffer distances), and green space quantification techniques. Street greenness promotes active travel by enhancing environmental esthetics, safety, and comfort, while also improving air quality, reducing noise, and fostering social interactions. In addition, the study suggests that variables like weather, seasonality, and cultural context may also correlate with the effectiveness of street greenery in encouraging active travel. To gain deeper insights into these complex relationships, future research should pivot toward experimental and longitudinal methodologies. Empirical analyses focusing on the intermediary mechanisms and contextual factors influencing the impact of street greenery on active travel are recommended. Such research approaches can elucidate the multifaceted dynamics of street greenness and its role in shaping active travel behavior more comprehensively.

## Data availability statement

The original contributions presented in the study are included in the article/supplementary material, further inquiries can be directed to the corresponding author.

## Author contributions

JY: Investigation, Writing – original draft, Software, Supervision, Validation, Writing – review & editing. HZ: Supervision, Writing – review & editing, Investigation, Validation. XD: Writing – review & editing, Supervision. JS: Writing – original draft, Writing – review & editing, Data curation, Funding acquisition, Investigation, Resources, Supervision.
